# Toxicological and Physiological Responses to Combined Ultrasound and Lipid-Coated ZnO Nanoparticle Exposure in *Caenorhabditis elegans*

**DOI:** 10.1021/acsami.6c07340

**Published:** 2026-06-16

**Authors:** Elia Pascucci, Giorgia Savino, Pamela Santonicola, Giuseppina Zampi, Elia Di Schiavi, Valentina Cauda

**Affiliations:** † Department of Applied Science and Technology, 19032 Politecnico di Torino, Corso Duca degli Abruzzi 24, 10129 Turin, Italy; ‡ Institute of Biosciences and BioResources (IBBR), National Research Council of Italy (CNR), Via P. Castellino 111, 80131 Napoli, Italy

**Keywords:** Nanomedicine, Zinc oxide nanoparticles, Acoustic stimulation, *Caenorhabditis elegans*, *In vivo* toxicity

## Abstract

In the field of nanomedicine, nanoparticles have gained increasing attention due to their potential as therapeutic and diagnostic tools across a wide range of biomedical applications. However, despite their growing popularity and promising results, understanding their biological interactions and accurately evaluating their safety and efficacy remain significantly challenging. In this context, the invertebrate model organism *Caenorhabditis elegans* (*C. elegans*) provides an ideal toxicological model system because of its favorable characteristics. In this research, we used *C. elegans* to investigate the effects of potentially toxic iron-doped zinc oxide nanoparticles (ZnO NPs) and their biocompatible counterpart, lipid-coated ZnO (L-ZnO NPs). In addition, we combine these NPs with physical stimulation, i.e., ultrasound, to which these NPs are responsive, aiming to evaluate possible combined treatments in animals. The toxicity of ZnO and L-ZnO NPs was evaluated on this invertebrate model. In addition, external acoustic pressure stimulation was studied, evaluating the sole effects of ultrasound stimulation and its combined application with NPs. Multiple biological parameters were analyzed to assess treatment effects, including viability, biodistribution, egg-laying, body bends, and production of radical species associated with oxidative stress. This study demonstrates that L-ZnO NPs exhibit greater biological safety than ZnO NPs, while their combined application with ultrasound does not result in an additive effect. Additionally, the results highlight the potential of using *C. elegans* as a primary model to evaluate nanomedicine treatments and obtain initial insights into the effects of nanoparticles in animal systems.

## Graphical abstract

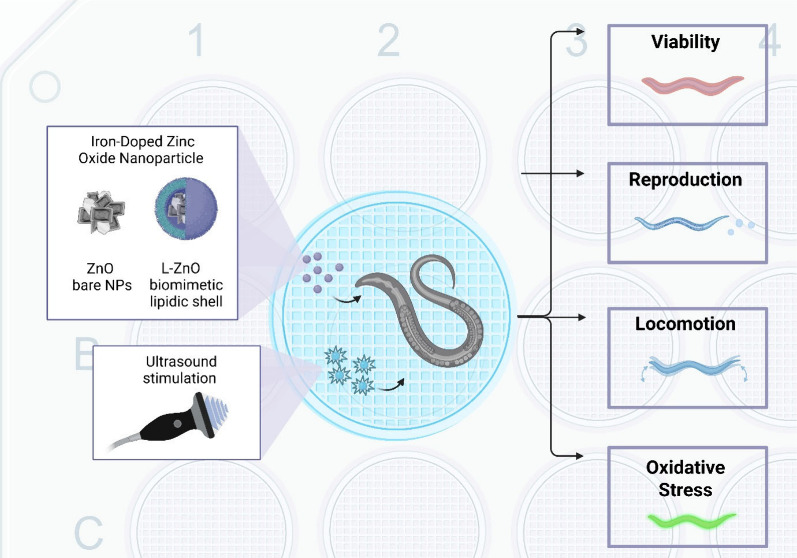

## 1 Introduction

Over the past few years, advantages in nanotechnology and deeper insight into nanoscale structure have significantly influenced numerous industrial sectors, particularly the field of nanomedicine.^1^ Nanomedicine aims to deliver therapeutics or bioactive compounds to specific target sites for treatment of various diseases. Beyond therapeutic applications, nanotechnology also enables the incorporation of diagnostic functionalities, allowing real-time monitoring of disease progression and the treatment response.^2^ The integration of diagnostic and therapeutic capabilities into a single nanoplatform—commonly referred to as theranostics—has become a key objective in the field,^3^ allowing one to obtain simplified yet highly effective systems. Nanoparticles (NPs) are especially well-suited for this purpose, as their tunable physicochemical properties allow them to serve simultaneously as therapeutic carriers and diagnostic agents. Owing to these capabilities, nanoparticles have been extensively investigated and rapidly developed for potential clinical applications.^4^ Importantly, the therapeutic capabilities of these nanoparticles can be triggered and activated on demand by energetic stimulation, such as light, magnetic fields, and acoustic waves.^5,6^ Among inorganic NPs, zinc oxide nanoparticles have shown numerous advantages,^7,8^ as promising theranostic agents, including biocompatibility, biostability, and antimicrobial activity.^9–11^

Within this conceptual framework, our group has recently developed iron-doped ZnO nanoparticles as a multifunctional nanoplatform, for simplicity, hereafter referred to as ZnO NPs. These nanostructures were further functionalized through self-assembly with a custom-made lipidic shell to improve colloidal stability, biocompatibility, and biological interfacing, thereby yielding an integrated theranostic platform specifically tailored for oncological applications.^12^ These NPs were coupled with remote mechanical stimulation to demonstrate their role as a sonosensitizer agent. For this purpose, we have chosen ultrasound stimuli (US), already applied in clinics to treat muscle-skeletal diseases to enable on-demand cell death for a controlled and safe therapy.

In relation to the current literature on this topic, acoustic stimuli can induce inertial cavitation, which is a process that generates reactive oxygen species (ROS) and mechanical stress. This effect is enhanced in the presence of nanoparticles, which can act as cavitation nuclei by stabilizing gas pockets on their surfaces or within their structure. Consequently, combining nanoparticles with US lowers the cavitation threshold and facilitates cavitation-related phenomena.^13–15^ Cytotoxicity studies suggest that all of these phenomena, enhanced by Zn^2+^ ion release and membrane permeabilization, contribute to damage cells.^16,17^

Further preclinical studies of our group investigated the use of this synergic treatment in both 2D culture of colorectal tumor cells and then 3D tumor models including spheroids and tubular structures.^18,19^

We feel that it is needed to perform *in vivo* studies on healthy animals to evaluate the intrinsic toxicity of ZnO NPs without drug, on the intrinsic toxicity of acoustic pressure waves, and finally the possible combinatorial use (NPs and US) to understand any interaction, positive or negative (i.e., ROS production, heating), paving the way for a responsible and safe use for future sonodynamic treatments.

For this purpose, we have chosen *Caenorhabditis elegans* (*C. elegans*) as an alternative *in vivo* model for toxicology screening of nanoparticles. *C. elegans* is a transparent, free-living soil nematode with a fully sequenced genome and a completely defined cell lineage. Its short life cycle, characterized genome, and ease of maintenance have contributed to its use in toxicology studies.^20^ In previous works, the nematode *C. elegans* has been widely employed as a screening platform for nanomaterials,^21^ offering a rapid and cost-effective means to investigate nanoparticle interactions at the molecular, cellular, and behavioral levels and investigate the full span of life of the animal.^22–24^ This model has been used to assess the toxicity of zinc oxide nanoparticles in various contexts, including environmental toxicity,^25^ aging-related processes,^26^ germ cell apoptosis and instability,^27,28^ and oxidative stress induction.^29^ Although the mechanism by which the pharynx pumps, transports, and filters food particles is not yet completely understood, the pharynx and the buccal cavity efficiently catch and transport particles of a size range corresponding to most bacterial strains (0.5–3 μm).^30^ Nevertheless, toxicity studies using *C. elegans* as a test organism have demonstrated that NPs can be efficiently taken up by the nematode both when mixed with a food source^31,32^ and when freely suspended in aqueous media.^33,34^ In recent years, *C. elegans* has also been employed as an *in vivo* model for investigating the biological effect of ultrasound stimulation.^35^ Several studies have explored how ultrasonic parameters, such as frequency,^36^ pulse repetition rate,^37^ duty cycle,^38^ temporal characteristics,^39^ and the presence of microbubble,^36,40^ affect worm behavior. Although previous results show that ultrasound can induce a neural response in the nematode, the underlying mechanism remains unclear.^36^ Further research is needed to unravel these mechanisms and fully understand the impact of ultrasound on *C. elegans*. In addition, emerging evidence is reported on the effect of nanoparticles on *C. elegans*, while almost no studies, to the best of authors’ knowledge, investigated the effect of a combined treatment, such as nanoparticles and physical stimulation, like light, magnetic fields, or ultrasound.

In this work, we aimed to investigate the biological effects of ZnO NPs using *C. elegans* as an *in* *vivo* model organism, which, owing to its well-characterized genetics, conserved molecular pathways, optical transparency, and suitability for high-throughput analysis, represents a powerful platform for nanotoxicological and mechanistic studies. Specifically, we evaluated the effects of bare (ZnO) or lipid-coated zinc oxide nanoparticles (L-ZnO), ultrasound stimulation, and their combination on key organism parameters, including viability, egg production, locomotion, and nanoparticle biodistribution, with the final aim to understand their systemic effect on healthy animal models, prior to using them for therapy for diseased models.

## 2 Materials and Methods

### 2.1 Preparation of ZnO NPs and Functionalization

ZnO NPs were synthesized by a wet chemical process, according to procedures previously described by our research group.^9^ Briefly, zinc acetate dihydrate (526 mg, ACS reagent, Sigma-Aldrich) was dissolved in 40 mL of absolute ethanol (99%, Sigma-Aldrich), which was then heated to 70 °C. Subsequently, 140 μL of oleic acid (≥99%, Sigma-Aldrich) and 1 mL of Milli-Q water (Millipore, Burlington, MA, USA) were added to the solution. 1.044 mg of tetramethylammonium hydroxide pentahydrate (TMAH, 98.5%, Sigma-Aldrich) was dissolved in 1.052 mL of Milli-Q water, and 10 mL of ethanol was then added to the solution after 10 min of stirring at 70 °C. After another 10 min of reaction, the ZnO NPs were centrifuged at 8000*g* for 10 min and washed three times with fresh ethanol.

The surface of the ZnO NPs was then functionalized with aminopropyl groups by adding 10 mol % 3-aminopropyltrimethoxysilane (APTMS, Sigma-Aldrich). Specifically, the NPs were resuspended in ethanol at a concentration of 2 mg/mL and heated to 70 °C with gentle stirring and nitrogen reflux. The APTMS was then dispersed within the solution, and the reaction was conducted for the next 6 h. At the end of the procedure, the NPs were washed by centrifugation three times at 12,000*g* for 20 min. For the incubation test, ZnO NPs were further centrifugated at 14,000*g* for 10 min and subsequently resuspended in Milli-Q water to achieve the desired concentration.

### 2.2 NP Coating with Lipid Bilayer

To improve biocompatibility, ZnO NPs have been coated with a lipid shell previously developed by our group.^12^ In particular, a mixture of DSPE-PEG(2000)-amine (1,2-distearoyl-*sn*-glycero-3-phosphoethanolamine-*n*-[amino­(polyethylene glycol)-2000], from Avanti Polar Lipids), DOPA (1,2-dioleoyl-*sn*-glycero-3-phosphate, from Avanti Polar Lipids), DOPC (1,2-dioleoyl-*sn*-glycero-3-phosphocholine, from Avanti Polar Lipids), and cholesterol (Sigma-Aldrich) in a ratio of 1.5:50:10:38.5 was dried under vacuum overnight. Subsequently, the lipids were resuspended in a mixture of absolute ethanol and Milli-Q water in a ratio of 40:60, reaching a final concentration of 3 mg/mL. The resulting lipid mixture was then added to ZnO NPs, previously pelleted at 14,000*g* for 10 min, in a NP:lipid weight ratio of 2:1. After 3 min of sonication at 59 kHz using an ultrasonic bath (Branson 3800 CPXH), a volume of Milli-Q water was added to reach a final concentration of 1 mg/mL. A further sonication for 5 min was then applied to improve sample homogenization. Following the preparation of the lipidic-shell ZnO NPs (L-ZnO NPs), the particles were diluted in Milli-Q water to the desired concentration for use in the incubation experiments.

### 2.3 NP Characterization

Z-potential and hydrodynamic radius measurements were performed with a Zetasizer Nano ZS90 (Malvern Instruments). Measurements were performed for both formulations in Milli-Q water at a concentration of 100 μg/mL.

The ZnO nanocrystals were characterized by high-resolution transmission electron microscopy (HR-TEM). The nanocrystals were dispersed in water at a concentration of 50 μg/mL. Subsequently, 10 μL was deposited on a lacey carbon substrate (300 mesh, Cu, Ted Pella Inc.) and allowed to dry.

Subsequent measurements were performed with a Thermo Scientific Talos F200X G2 S­(TEM) at an operating voltage of 200 kV for bare ZnO and reduced to 60 kV for ZnO-Lip.

### 2.4 *C. elegans* Maintenance and Strains

Nematodes were grown and handled following standard procedures^41^ on nematode growth medium (NGM) agar plates seeded with *Escherichia coli* strain OP50. In this work, all experiments were conducted with the wild-type N2 strain, except for the ROS quantification assay, which was performed using a transgenic strain ubiquitously expressing the H_2_O_2_ sensor HyPer: JV1 [*unc-119­(ed3); jrls1­(rlp-1p::Hyper)*].^42^ Strains used in this work have been provided by the *Caenorhabditis* Genetics Center (CGC).

### 2.5 NP Toxicity *in Vivo* Assays in *C*. *elegans*

Acute exposure experiments were performed in liquid using 96-well plates, with treatments consisting of ZnO NPs, L-ZnO NPs, and Milli-Q water as control. About 20 adults were initially allowed to lay eggs for 2 h on each NGM plate to obtain synchronized eggs, and worms were cultured on the plates seeded with *E. coli* OP50 until they reached the L4 larval stage. Synchronized L4 larvae were then gently washed off the agar plates with Milli-Q water and collected by centrifugation at 1,300*g* for 1 min. Residual water was removed by carefully sucking the supernatant without touching the worm pellet, which was subsequently transferred to a NGM plate without a food source. This washing procedure was repeated two times to ensure the removal of as much bacterial residue as possible. After the washing steps, approximately 30 L4-stage animals were transferred into each well for the liquid exposure assays. Worms were incubated overnight with the NPs for analyzing the viability, the lifespan, the brood size, and the biodistribution. Worms were incubated with the NPs for 2 h for the locomotion assay (see Figure S1). Following exposure, worms were transferred to fresh NGM plates seeded with *E. coli* OP50 for recovery and further analysis.

#### Viability Test

An initial acute exposure experiment with food was performed using synchronized L4-stage *C. elegans* (see Section 2.5), testing ZnO and L-ZnO nanoparticles with concentrations of 50, 100, and 200 μg/mL, and Milli-Q water was used as mock. Worms were treated in 70 μL of a mix containing M9 buffer (3 g KH_2_PO_4_, 6 g Na_2_HPO_4_, 5 g NaCl, 1 mL MgSO_4_ 1 M, Milli-Q water up to 1 L) supplemented with 2× antibiotic/antimycotic solution (Sigma-Aldrich, cat. A5955), 5 ng/mL cholesterol (Sigma-Aldrich, cat. C8667), and *E. coli* OP50 as a food source.^43,44^ All subsequent experiments were conducted without a food source,^45^ incubating worms in a 70 μL mix of Milli-Q water as mock and ZnO or L-ZnO nanoparticles with concentrations of 0.5, 1, 10, 20, 30, 50, 100, and 200 μg/mL. After exposure, worms were recovered, and the number of living animals was quantified per replicate, with a minimum number of replicates corresponding to three. The percentage of viable animals for each concentration was calculated using the total number of animals present on the plate.

#### Lifespan Assay

For the lifespan assay, synchronized animals were treated in liquid, as described above, with ZnO and L-ZnO NPs at a final concentration of 30 μg/mL and Milli-Q water as mock. After the treatment, 100 adult animals for each condition were transferred onto 20 fresh NGM plates without treatment (5 animals for each plate). The viability of the animals was scored every 2 days, and animals were transferred every 2 days to fresh plates. Animals that crawled off the plate were censored.

#### Brood Size Assay

To test the NPs’ effect on brood size, animals were treated in liquid as described above, with ZnO and L-ZnO NPs at a final concentration of 30 and 100 μg/mL and Milli-Q water as mock. After the treatment 20 animals per treatment were transferred to a fresh plate without treatment. Then, every 24 h animals were moved to new plates without any treatment for all their fertile period (4 days) and the total number of laid eggs was counted every day. After the treatment, the animals were also examined, and those presenting vulva defects were counted. Images were collected with a Zeiss microscope Axio Imager KMAT.

#### Locomotion Assay

To evaluate *C. elegans*’ locomotion after incubation with nanoparticles, animals were treated at different concentrations of 0.5, 10, 30, and 100 μg/mL ZnO and L-ZnO NPs, as described above. Mobility was assessed by body bend analysis. For this purpose, agar plates were prepared with a central drop of *E. coli* OP50. Following the incubation, the animals were recovered by centrifugation at 375*g* for 5 min and placed on a new agar plate with *E. coli* OP50. After drying from residual water, worms were transferred within the drop of bacteria to a new agar plate. After 80 s of acclimation, a 20 s video was captured using a Leica Flexacam i5 video camera to assess body bend movements.

#### Biodistribution

ZnO and L-ZnO NPs were labeled prior to the lipid coating with ATTO-647-NHS ester (Sigma). 10 μL of a 2 mg/mL solution was added for each mg of NPs to a suspension of ZnO NPs in ethanol at 1 mg/mL concentration and stirred overnight at 250 rpm at room temperature. Then, the ZnO NPs were washed by multiple centrifugations (10 min at 14,000*g*) followed by resuspension steps in ethanol. After the last centrifugation, the pellet was either resuspended in Milli-Q water (ZnO) or coated by the lipidic formulations (as described in Sections 2.3 and 2.4) and resuspended (L-ZnO), to obtain a ZnO NP concentration of 1 mg/mL. The final suspension was sonicated for 10 min. Animals were recollected after an incubation in liquid for 2 h or overnight with labeled ZnO and L-ZnO NPs at a concentration of 100 μg/mL and Milli-Q water as mock. Animals were transferred to fresh NGM with *E. coli* OP50 and allowed to clean from excessive dye for 2 h. They were transferred on glass slides with 4% agar pads and immobilized alive for microscopy analysis with 0.01% tetramisole hydrochloride (cat. no. T1512 Sigma-Aldrich). The images were collected with a Leica TCS SP8 AOBS confocal microscope using a 60× objective and the following settings: laser intensity 10; excitation 630 nm; emission 650–750 nm.

### 2.6 Ultrasound Experimental Setup

Ultrasound (US) exposure experiments were performed in liquid for viability assays and in solid for locomotion assays. For the ultrasound stimulation we used a 2 cm^2^ US transducer (Chattanooga Intelect Transport Ultrasound, DJO LLC) coupled with an acoustic water-based gel (Stosswellen Gel, Elvation Medical GmbH) to ensure acoustic impedance matching. For the in-liquid treatment a temperature test was carried out to analyze the increase in temperature after the US treatment and the heating of nearby wells. In a well filled with 2 mL of water at room temperature (26 °C), the ultrasound at a power density of 0.5, 0.7, 1, and 2 W/cm^2^ was conducted for 1 min and the increase of temperature was measured in the treated well and the adjacent ones. We observed an increase in temperature only in the nearest well, and for this reason at least one empty well was left in each direction to avoid any influence of temperature of the ultrasound treatment on the phenotype analyzed.

#### Viability Test

*C. elegans* animals were synchronized and cleaned from bacteria as described in Section 2.5. Approximately 50 L4 larval stage animals were transferred into each well of 24-multiwell plates for the liquid exposure assays. Worms were incubated overnight in 1 mL of Milli-Q water. The following day, each well containing worms was filled with water at 15 °C and closed with an adhesive sealing film (Thermo Scientific, 15036, nonsterile sealing tape for 96-well plates). The well was positioned in the central part of the Chattanooga transducer by interposing a thin layer of gel. Different US settings, maintaining a frequency of 1 MHz, were evaluated (0.3, 0.5, 0.7, 1, and 2 W/cm^2^) for 1 min, and nontreated wells were considered as mock. Following exposure, worms were collected by centrifugation at 375*g* for 5 min and then transferred to fresh NGM plates seeded with *E. coli* OP50 for recovery. The number of living animals was quantified per replicate, with a minimum number of replicates corresponding to three. The percentage of viable animals for each treatment was calculated on the total number of animals present on the plate.

#### Locomotion Assay

To assess motility after ultrasound exposure, L4-stage worms were transferred from NGM plates seeded with *E. coli* OP50 to new NGM plates containing a single central drop of *E. coli* OP50. After 80 s of acclimation, the worms were subjected to ultrasound treatment at different power densities, specifically 1 and 2 W/cm^2^, for different time intervals (1, 2, and 3 min). To treat the animals, the ultrasound probe was placed in contact with the bottom of the Petri dish separated by a thin layer of gel and centered beneath the drop of bacteria containing the worms. After the treatment, we waited 80 s before video acquisition for 20 s.

### 2.7 Combined Treatment Experimental Setup

A treatment combining NPs and US exposure was carried out in liquid in 24-well plates to evaluate the toxicity of the combinatorial treatment.

#### Viability Assay

*C. elegans* animals were synchronized and cleaned from bacteria as described in Section 2.5. Approximately 50 L4 larval stage animals were transferred into each well of 24-multiwell plates for the liquid exposure assays. Worms were incubated overnight in 1 mL of L-ZnO at a concentration of 100 μg/mL and in Milli-Q water as mock. The following day each well containing worms was filled with water at 15 °C and closed with adhesive sealing film. The well was positioned in the central part of the Chattanooga transducer by interposing a thin layer of gel. Different US settings, maintaining a frequency of 1 MHz, were evaluated (0, 0.3, 0.5, 0.7, 1, and 2 W/cm^2^) for 1 min. Following exposure, worms were transferred to fresh NGM plates seeded with *E. coli* OP50 for recovery and the number of living animals was quantified per replicate, with a minimum number of replicates corresponding to three. The percentage of viable animals for each concentration was calculated on the total number of animals present on the plate.

#### Locomotion Assay

To evaluate the effects of ultrasound treatment on the motility of animals previously incubated with nanoparticles, 50 L4 larval stage worms were incubated for 2 h with 30 μg/mL of ZnO NPs or with 100 μg/mL of L-ZnO NPs in a total volume of 1 mL in 24-multiwell plates. Following treatment with NPs, the wells were filled with water at 15 °C and closed with adhesive sealing film. The wells were then treated with different power densities (0.5, 0.7, 1, and 2 W/cm^2^) for 1 min, from the bottom of the well. Following the combined treatment, worms were collected and allowed to dry on an NGM plate with *E. coli* OP50. Once dried, they were analyzed by moving them to plates containing only a single drop of *E. coli* OP50 in the center of the NGM plate. After waiting 80 s for the animals to recover, a 20 s video was acquired to evaluate body bends.

#### ROS Measurement *in Vivo*

To evaluate the production of reactive oxygen species (ROS), the transgenic *C. elegans* strain JV1 expressing the H_2_O_2_-sensitive HyPer sensor was used. The effects of L-ZnO incubation alone, ultrasound stimulation alone, and their combination were evaluated. Approximately 50 L4 larval stage worms were incubated in 24-well plates with 1 mL of L-ZnO at a concentration of 100 μg/mL and Milli-Q water as mock for 2 h. Following treatment with NPs, the wells were filled with water at 15 °C and closed with adhesive sealing film. The ultrasound treatment was carried out for the required groups for 1 min with a power density of 2 W/cm^2^. Following exposure, worms were collected with a 375 g centrifugation for 5 min and subsequently transferred to fresh NGM plates seeded with *E. coli* OP50 for recovery. For observation at the microscope, five animals were placed on slides prepared with a 4% agar pad and immobilized with 3 μL of 30 mM sodium azide (NaN_3_, Sigma-Aldrich) added to the pad. The sample was then covered with a coverslip, and a collection of images was acquired using a Leica TCS SP8 AOBS inverted microscope using a 20× objective, equipped with an FITC filter. The intensity of the Hyper signal was then quantified using ImageJ. Fluorescence intensity was quantified using corrected total cell fluorescence, calculated as the integrated density minus the product of the area of interest and the mean background fluorescence.

### 2.8 Figures

All of the graphs were created in GraphPad Prism 10, and all of the schemes were produced with BioRender.com.

### 2.9 Statistical Analysis

Statistical analyses were performed with the GraphPad Prism 10 software. The experimental replicates, the sample size used to derive statistics (*n* = number of biologically independent animals), and statistical analyses used for each experiment are described in the figure legends. Differences were accepted as significant when *p* < 0.05.

## 3 Results and Discussion

### 3.1 Nanoparticle Characterization

Toxicity of zinc oxide nanoparticles was assessed by exposing *C. elegans* to two types of nanoparticles: zinc oxide nanoparticles (ZnO) and lipid-coated zinc oxide nanoparticles (L-ZnO). To reduce the possible toxicity of ZnO NPs and increase the biocompatibility and stability, as well as their biomimetic characteristics, ZnO NPs were coated by a lipidic formulation composed of regular and anionic phospholipids, cholesterol, and PEGylated phospholipids, following previous work of our group.^12^ A solvent exchange method was employed, exploiting the electrostatic interaction among the positively charged bare NPs and the overall negative charge of the lipid mixture. After hydration, the lipid mixture self-assembles and forms a thin bilayer around clusters of nanoparticles. The morphologies of the ZnO and L-ZnO NPs dispersed in water were determined by the analysis of the TEM images, while their size and zeta potential values were analyzed to verify the effectiveness of the lipid coating. The morphology of ZnO was evaluated by HR-TEM. Figure 1A highlights the typical crystalline planes of zinc oxides in bare nanocrystalline particles. For L-ZnO, the images were obtained by low-voltage TEM (60 kV) on freshly prepared samples to preserve the lipid component. A halo due to the lipid coating can be observed around the zinc oxide nanocrystals (Figure 1B). From the dynamic light scattering (DLS) characterization, the hydrodynamic diameter of the bare nanoparticles in water is 118.3 nm with a PDI of 0.18 (Figure 1C, black line), whereas L-ZnO NPs show a hydrodynamic size of 187.2 nm with a PDI of 0.18 (Figure 1C, purple line). Both results show that the size distribution is uniform and relatively monodisperse. The Z potential value (Figure 1D) of the bare ZnO NPs is highly positive (30.1 mV), while, after the lipid coating with mainly negatively charged lipids, the Z-potential becomes −14.9 mV. This result demonstrates the effective coating of the lipidic shell on the nanocrystals, as also indicated in our previous work.^12,13,18,19^

**Figure 1:**
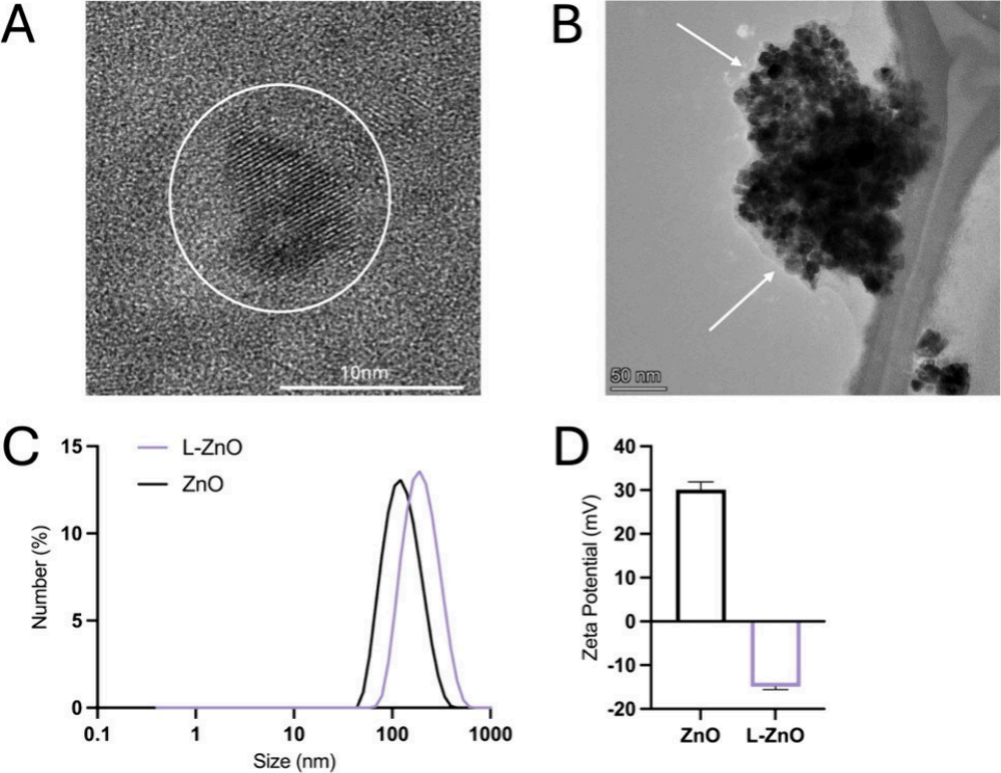
ZnO and L-ZnO characterization. (A) TEM images of ZnO (white circle indicates the crystallographic planes of the ZnO crystalline nanostructure) and (B) L-ZnO NPs (white arrows indicate the lipidic shell around a cluster of nanoparticles). (C) DLS percentage measurement and (D) Z potential of ZnO and L-ZnO in water at a concentration of 100 μg/mL. Error bars represent standard deviation.

### 3.2 ZnO and L-ZnO NP Toxicity Assays

To test the effect *in vivo*, we evaluated the response of *C. elegans* animals treated in liquid with nanoparticles. In the present study, a first experiment was conducted to investigate the dose-dependent toxicity effect of these nanoparticles. Initially, worms were incubated overnight in liquid with *E. coli* OP50 as a food source across different test concentrations of ZnO and L-ZnO NPs (50, 100, and 200 μg/mL). These concentrations were chosen following previous experiments conducted by our group on both 2D cell monolayers and 3D models.^18,19^ Despite the relatively high concentration of NPs, an unexpected high viability was observed for all the tested concentrations (Figure S2.A). This can be possibly explained with the notion that *E. coli* may absorb,^33^ aggregate, or metabolize and dissolve an unknown portion of the tested NPs,^46^ thereby decreasing the effective concentration of nanoparticles in suspension. This could reduce exposure levels, alter ion release from the nanoparticles and affect their biodistribution and excretion. For this reason, a second experimental setting was used in the absence of bacterial food.^45^ An overnight exposure of L4-stage larvae to ZnO NPs in absence of *E. coli* OP50 caused animal mortality, which increased by increasing concentration (Figure 2A). An LC_50_ of 27 μg/mL was determined by nonlinear regression (Figure S2B). Importantly, we did not observe any mortality in animals treated with L-ZnO, similarly to the animals treated with mock, thus indicating the high biocompatibility of L-ZnO in contrast to their naked counterpart. These results are consistent with data previously obtained in 2D cell-based cytotoxicity assays and support the hypothesis that the higher toxicity observed for ZnO nanoparticles may be related to the absence of the lipid coating, which could exert a protective effect by reducing direct nanoparticle interactions and Zn^2+^ release. Therefore, we decided to use concentrations of 30 and 100 μg/mL as the optimal doses to further evaluate nanoparticle effects on lifespan, reproduction, and motility. Since the two nanoparticle formulations showed different toxicity profiles, the concentrations were selected independently for each formulation in order to maximize exposure while maintaining viability above 50%.

**Figure 2:**
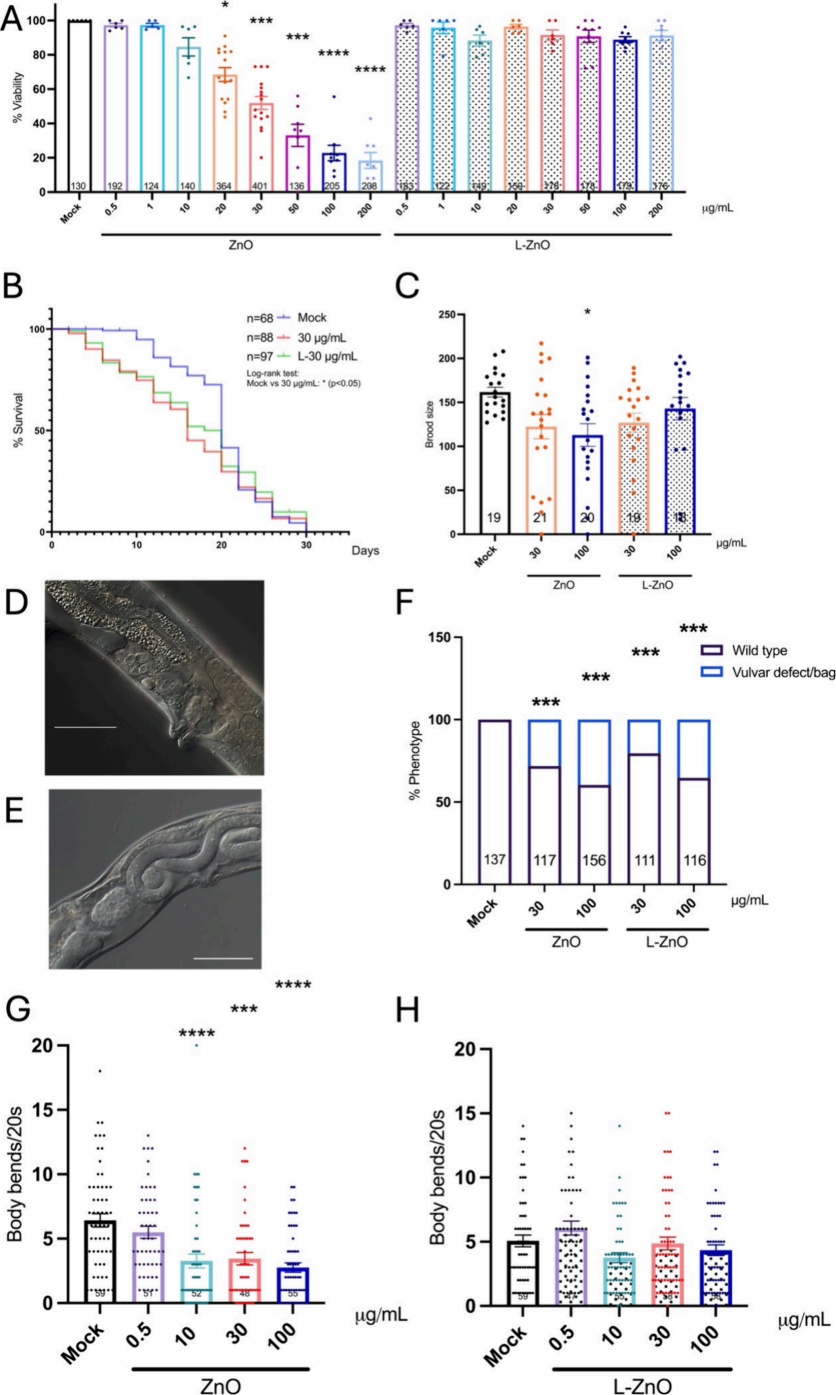
Evaluation of the effect of ZnO and L-ZnO NPs. (A) Quantification of the percentage of living animals after overnight exposure to ZnO and L-ZnO NPs (0.5, 1, 10, 20, 30, 50, 100, 200 μg/mL) in absence of bacterial food source. Exposure of NPs was performed from L4 larval stage animals. Statistical significance of the differences between treatments with mock and nanoparticles was assessed with Kruskal–Wallis one-way ANOVA: *p < 0.05; ***p < 0.0005; ****p < 0.0001. Each dot corresponds to the percentage of living animals for replicate. Bar is the mean value. Error bars indicate SEM. (B) Lifespan of animals treated with mock (blue line), ZnO (red line), and L-ZnO NPs (green line) at a concentration of 30 μg/mL. Statistical significance of the differences between treatments with mock and nanoparticles was assessed with a log-rank (Mantel–Cox) test. ZnO NPs showed a statistically significant reduction in survival compared to mock (*p < 0.05), while L-ZnO showed no significative difference. (C) Brood size of animals after treatment with mock, ZnO, and L-ZnO NPs at a concentration of 30 and 100 μg/mL. Statistical significance of the differences between treatments with mock and nanoparticles was assessed with Kruskal–Wallis one-way ANOVA: *p < 0.05; ns > 0.05. Each dot corresponds to the total number of eggs laid by one worm in 4 days. Bar is the mean value. Error bars indicate SEM. (D) Image of the vulva of *C. elegans* after treatment with L-ZnO NPs. (E) Image of *C. elegans* bagged after treatment with ZnO NPs. Scale bars: 50 μm. (F) Percentages of animals presenting vulva defects after treatment with ZnO and L-ZnO NPs (30 and 100 μg/mL). Statistical significance of the differences between treatments with mock and nanoparticles was assessed with two-proportion nonparametric Z-test. ***p < 0.0005. (G, H) Quantification of the body bends performed by animals in 20 s after 2 h incubation without food at different concentrations (0.5, 10, 30, and 100 μg/mL) of (G) ZnO and (H) L-ZnO NPs. Statistical significance of the differences between treatments with mock and nanoparticles was assessed with Kruskal–Wallis one-way ANOVA: ns > 0.05; **p < 0.0005; ****p < 0.0001. Each dot corresponds to the number of body bends in 20 s made by the worm. Error bars indicate SEM. In A, C, G, and H the numbers in the columns correspond to the number of animals tested.

Specifically, for ZnO nanoparticles, a concentration of 30 μg/mL was chosen, as this concentration was closest to the LC_50_ value (51.87% viability). In contrast, for L-ZnO nanoparticles, the viability remained consistently high; therefore, a higher concentration (100 μg/mL) was selected.

The differences observed in the two experimental settings with or without bacteria suggest that the presence of bacteria modulates the way nematodes respond to ZnO NPs. Thus, to further investigate the toxicity and the uptake of ZnO and L-ZnO NPs in all the following experiments, L4 stage worms were incubated in Milli-Q supplemented with nanoparticles in the absence of a food source.

Following the viability assays, a biodistribution analysis was performed to assess nanoparticle uptake in *C. elegans*. Since only L-ZnO NPs did not affect viability, we decided to assess their biodistribution and evaluate their internalization. In *C. elegans*, ingested particles typically pass through the pharynx and then accumulate in the intestinal lumen, one of the major organs responsible for food digestion and assimilation and synthesis and storage of macromolecules.^47^ As shown in Figure S3A, the fluorescent signals from both ZnO and L-ZnO NP samples were detected within the intestinal lumen, with a stronger intensity beyond the pharynx. In contrast, all the mock animals treated with the same experimental conditions exhibited no fluorescence (Figure S3B). Notably, while both nanoparticle types were visible in the lumen, only L-ZnO NPs accumulated within the intestinal cells (Figure S3C). ZnO nanoparticles are known to undergo partial dissolution in biological media, leading to the release of Zn^2+^ ions, which are considered a major contributor to their toxicity. Conversely, the lipid coating of L-ZnO nanoparticles may reduce direct particle–intestinal interactions and limit Zn^2+^ release, thereby modifying both biodistribution and consequently their toxicity.^48^

To evaluate the biocompatibility of these nanoparticles, we assessed both the lifespan and the reproductive capacity of *C. elegans* animals after treatment (Figure 2B,C). For the lifespan assay, only 30 μg/mL of ZnO and L-ZnO NPs were tested. Both treatments caused an increased mortality during the first days of the experiment; however, ZnO NPs had the most pronounced effect, showing a statistically significant difference compared with the control. Furthermore, we examined the egg-laying ability and observed that all treated animals displayed some degree of alteration, with a broad variability and several worms producing either zero or few eggs. Among all tested conditions, only 100 μg/mL ZnO NPs caused a statistically significant reduction in egg production compared with the control. These findings indicate that exposure to ZnO nanoparticles negatively affects reproductive output in *C. elegans*. To better understand the reduced egg production, we examined the animals by microscopy analysis after treatment, and we observed vulval defects, such as a protruding vulva (Figure 2D), which is known to impair egg-laying. The observed phenotype also led to the accumulation of hatched animals inside the mother, known as bag of worms (Figure 2E). The number of animals presenting these phenotypes was counted and plotted in Figure 2F, with all treated groups that differed significantly from the control. Both the reduction in egg production and the increased occurrence of vulvar defects are considered indicators of reproductive and developmental toxicity. Both signals suggest that even the coated nanoparticles, although not lethal, still exhibit a degree of toxicity.

Finally, we analyzed the effect of the NPs on the motility of *C. elegans* using the body bend assay. As shown in Figure 2G, incubation with uncoated ZnO NPs resulted in a significant reduction in the number of body bends performed in 20 s compared to the control, starting from a concentration of 10 μg/mL. This effect did not increase at a concentration higher than 10 μg/mL. The decrease in body bends may be connected to a possible toxic effect of NPs, thus suggesting a possible alteration of the animal’s neuromuscular system. In conclusion, this result, together with those previously shown, suggests that the exposure to ZnO NPs compromises viability, fertility and locomotor functions. Figure 2H shows the effect of L-ZnO NPs on motility. In this case, the incubation did not cause a significant change in the number of body bends at increasing concentrations compared with the mock group. Considering this and the previous data, incubation with L-ZnO NPs did not show any significant effect on viability, fertility, and motility.

### 3.3 Ultrasound Effect

We first focused on determining the effect of ultrasound treatment alone on *C. elegans*. L4 larval stage animals were left overnight in liquid without a food source; then they were exposed to ultrasound, recollected, and analyzed. Worms were exposed to increasing ultrasound power densities for 1 min. The viability (Figure 3A) remained consistently high at intensities up to 0.7 W/cm^2^, suggesting that these levels of US are well tolerated. At 1 W/cm^2^, the viability became more variable, which may indicate the onset of physiological stress, and exposure to 2 W/cm^2^ resulted in a dramatically reduced survival, demonstrating that this intensity exceeds the tolerance limit of worms.

**Figure 3:**
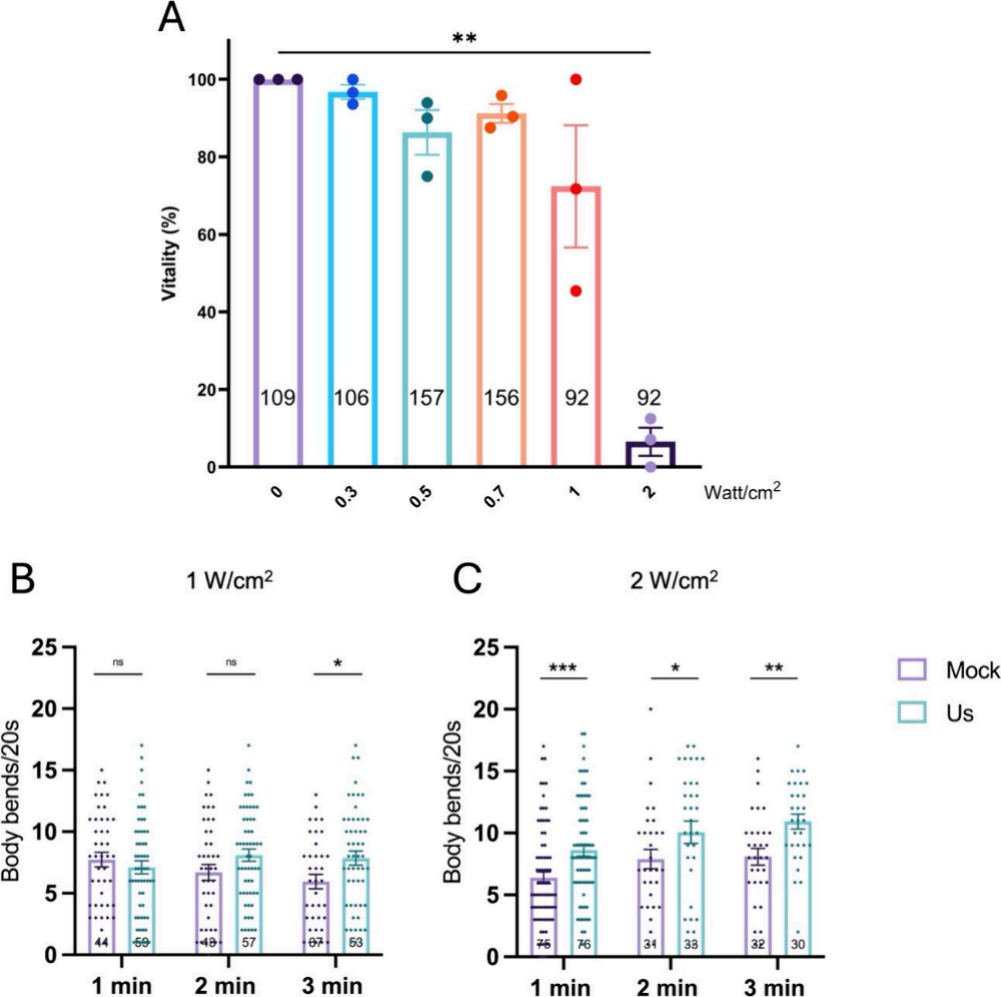
Evaluation of the effects of ultrasound treatment. (A) Quantification of the percentage of living animals after exposure to different ultrasound power densities (0, 0.3, 0.5, 0.7, 1, and 2 W/cm^2^). Statistical significance of the differences between treatments with mock and ultrasound was assessed with Kruskal–Wallis one-way ANOVA: **p < 0.01. Each dot corresponds to the percentage of living animals in each replicate. Bar is the mean value. Error bars indicate SEM. (B, C) Measurement of body bends in 20 s after different times (1, 2, and 3 min) of treatment at 1 W/cm^2^ (cyan in B) and 2 W/cm^2^ (cyan in C) compared to mock-treated animals (purple in B and C). Statistical significance of the differences between treatments with mock and nanoparticles was assessed with a Mann–Whitney *t* test: ns > 0.05; *p < 0.05; **p < 0.01; ***p < 0.001. Each dot corresponds to the number of body bends in 20 s made by the worm. Error bars indicate SEM. In A, B, and C the numbers in the columns correspond to the number of animals tested.

Body bends were quantified (Figure 3B,C) at two power densities (1 W/cm^2^ and 2 W/cm^2^) and for different treatment times (1, 2, and 3 min of stimulation) in an agar plate to visualize the response immediately after the treatment. At 1 W/cm^2^ (Figure 3B), we observed that 1 and 2 min treatments did not lead to a statistically significant difference in the number of body bends. A difference was observed when analyzing the animals after a 3 min stimulation. At 2 W/cm^2^ (Figure 3C), a statistically significant increase in the number of body bends was observed starting from 1 min of treatment compared to the control group. These results demonstrate the sensitivity of *C. elegans* to ultrasonic stimulation, resulting in increased mobility of the animal after the treatment. In particular, the increase in locomotor activity can be interpreted as neuromuscular activation in response to an external ultrasound stimulation. Notably, the US treatment was carried out both in water and in agar at the same intensities. While worms exposed in water showed increasing mortality at an intensity above 0.7 W/cm^2^, those on agar plates exhibited increased locomotion without showing mortality. These differences can be explained by the different propagation properties of ultrasound in a liquid environment and on agar. In water, ultrasound can be transmitted more efficiently, favoring the animal’s exposure to both mechanical and thermal effects. Conversely, ultrasound propagation through agar can attenuate the mechanical and thermal effects, resulting in a lower stimulation at the same nominal parameters set in the experimental setup. For these reasons, it was possible to expose worms to high-power densities for a prolonged period (e.g., 3 min) in agar. These observations underscore the importance of the propagation medium in interpreting the biological effects induced by the ultrasound.

### 3.4 Combined Treatment Effects

Since ZnO nanoparticles coated with a lipidic shell exhibited a markedly reduced toxicity across several biological parameters, including viability, lifespan, and reproduction, in contrast to their uncoated counterparts, we evaluated their effects of the combined treatment of NPs and ultrasound. For the viability assay, we selected a single coated nanoparticle concentration (L-ZnO 100 μg/mL), following the data previously obtained. Worms after being incubated with NPs were subsequently exposed to different ultrasound power densities. As shown in Figure 4A, viability outcomes were primarily driven by the ultrasound treatment. No significant statistical differences were observed between the worms exposed to ultrasound alone and those exposed to the combined ultrasound and nanoparticle treatment. This indicates that the combination of ultrasound and L-ZnO nanoparticles did not introduce additional toxicity beyond that observed with the ultrasound treatment.

**Figure 4:**
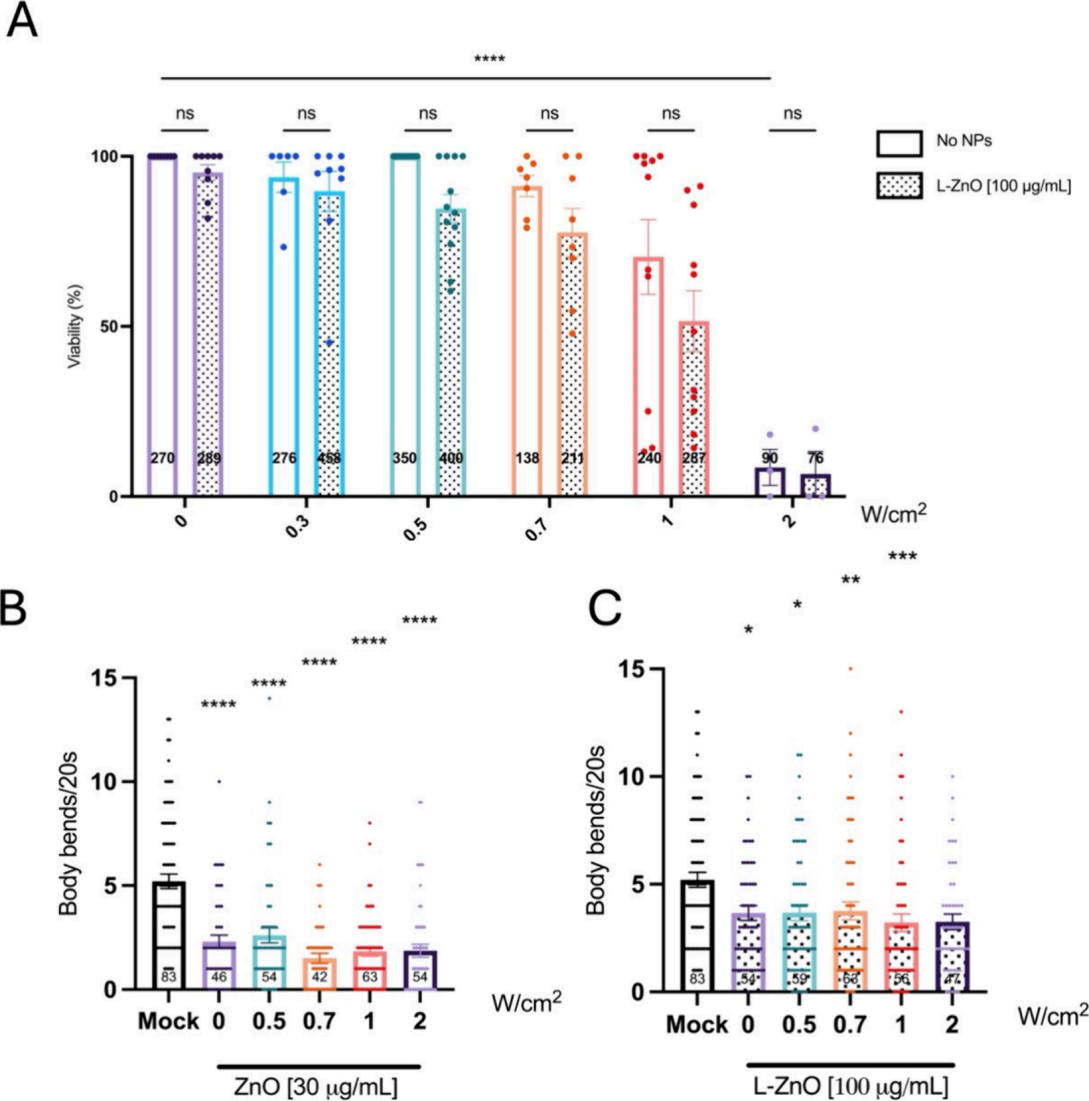
Evaluation of the effect of combined treatments. (A) Quantification of the percentage of living animals after incubation with L-ZnO NPs (100 μg/mL) and exposed to different ultrasound power densities (0, 0.3, 0.5, 0.7, 1, and 2 W/cm^2^). Statistical significance of the differences between treatments with and without NP incubation was assessed with two-way ANOVA multiple comparison test: ns > 0.05; ****p < 0.0001. Each dot corresponds to the percentage of living animals for each replicate. Bar is the mean value. Error bars indicate SEM. (B, C) Quantification of the body bends performed in 20 s after treatment with different ultrasound power densities (0, 0.5, 0.7, 1, and 2 W/cm^2^) following incubation for 2 h with (B) 30 μg/mL ZnO and (C) 100 μg/mL L-ZnO NPs. Statistical significance of the differences between treatments with mock and nanoparticles was assessed with Kruskal–Wallis one-way ANOVA: ns >0.05; *p < 0.05; **p < 0.01; ***p < 0.0005; ****p < 0.0001. Each dot corresponds to the number of body bends in 20 s made by the worm. Error bars indicate SEM. In A, B, and C the numbers in the columns correspond to the number of animals tested.

For the assessment of body bends, in contrast to the viability assay, both types of nanoparticles were tested. In particular body bends were analyzed following cotreatment with ZnO NPs (Figure 4B) at 30 μg/mL or L-ZnO NPs (Figure 4C) at 100 μg/mL, combined with different ultrasound power densities (0, 0.5, 0.7, 1, and 2 W/cm^2^). In both cases the combined treatment did not significantly alter the number of body bends compared to the group not stimulated by ultrasound, demonstrating that in this case the main effect is attributable to incubation with nanoparticles.

Since ZnO nanoparticles may induce oxidative stress in certain circumstances^49^ and ultrasonic stimulation can increase ROS production through inertial cavitation, we investigated whether the single and combined treatments could increase ROS levels in a transgenic *C. elegans* strain. To quantify ROS in whole animals, JV1 worms ubiquitously expressing the H_2_O_2_ sensor HyPer were treated, and the intensity of the fluorescence was used as an indicator of intracellular ROS accumulation. Specifically, four conditions were evaluated: control group, incubation with L-ZnO at 100 μg/mL, ultrasound exposure at 2 W/cm^2^ for 1 min, and the cotreatment. Representative images of the treated animals are shown in Figure 5A. Based on the quantitative evaluation of the fluorescence (Figure 5B), incubation with L-ZnO produced a statistically significant increase in the fluorescence signal compared to the control group, thus indirectly indicating an increase in ROS. A nonsignificant increase in ROS production is then observed after exposing the animals to the ultrasound treatment alone (2 W/cm^2^ for 1 min). Finally, a significant increase was observed by the combination of treatments compared to the control.

**Figure 5:**
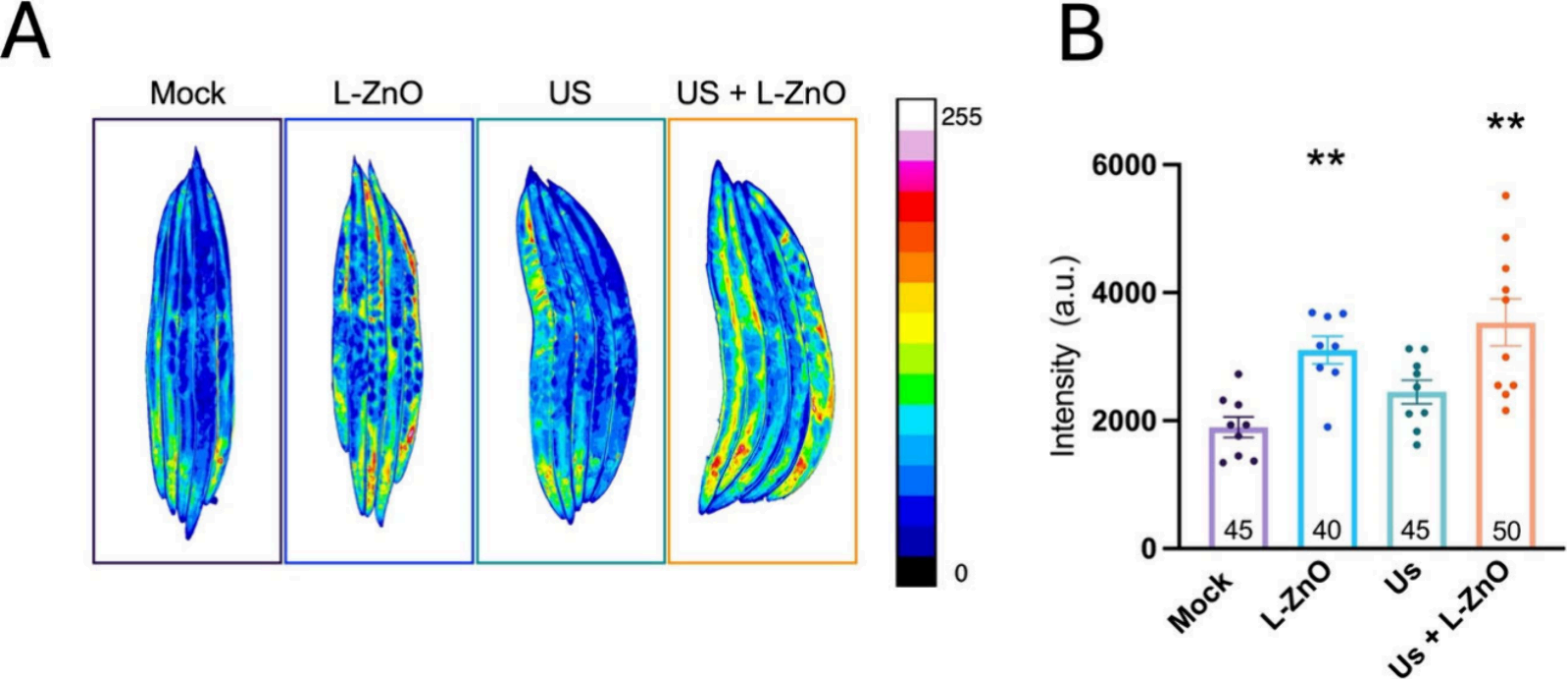
Evaluation of ROS production after combined treatments. (A) Representative images of 5 animals per group on a 16-color intensity scale, where white represents the highest signal intensity, corresponding to a pixel value of 255, whereas black represents the lowest signal intensity, corresponding to a pixel value of 0. (B) Quantitative evaluation of fluorescence intensity in transgenic worms subjected to different treatments: no-treatment (mock), 2 h incubation with L-ZnO NPs (100 μg/mL), ultrasound stimulation at 2 W/cm^2^ for 1 min (US), and combined treatments (US and L-ZnO). Each dot corresponds to a group of 5 animals. Error bars represent standard deviation. Statistical significance of the differences between treatments with mock and nanoparticles was assessed with Kruskal–Wallis test one-way ANOVA: **p < 0.01. In B the numbers in the columns correspond to the number of animals tested.

In summary, the data obtained by the combined treatment show how the mortality in *C. elegans* is primarily influenced by ultrasound stimulation, becoming statistically significant starting at 2 W/cm^2^. Preincubation with a previously identified safe concentration of NPs resulted in increased mortality at all tested power densities. However, under none of these conditions was the increase statistically significant, indicating that the combined treatment is statistically comparable to ultrasound exposure alone.

In contrast, the analysis of sublethal effects in surviving animals, specifically body bending and reactive oxygen species (ROS) production, revealed different trends. A reduction in locomotor activity and an increase in GFP fluorescence intensity were observed after incubation with lipid-coated nanoparticles alone. The same behavior in body bends was observed with the naked NPs. As highlighted previously, these results are consistent with the existing literature reporting that ZnO nanoparticles promote the generation of radical species.

These data therefore indicate that we find two independent mechanisms: NP incubation induces chemical stress through the generation of ROS, which also affects animal movement, while ultrasound induces primarily mechanical stress. In conclusion, we can state that the combined treatment does not appear to exert statistically significant additive effects, as measured by these healthy *in vivo* models.

## 4 Conclusions

In this study, we evaluated *C. elegans* for the toxicity and the effect on behavior of both bare and core–shell lipid-coated zinc oxide nanoparticles, with and without an ultrasound stimulation. We initially studied the phenotypes induced by the nanoparticle and ultrasound exposure and then in combination. We observed that *C. elegans* exposed to ZnO NPs exhibited increased mortality as the concentration increased, whereas animals treated with L-ZnO NPs did not show any lethal effects. Then we assessed the effective uptake of the nanoparticles by the animals, and we focused on different phenotypes associated with nanoparticle exposure. Notably, both NPs induced specific defects in vulval morphogenesis, suggesting that nanoparticle internalization may interfere with the developmental process. However, incubation of *C. elegans* with L-ZnO NPs indicated higher biocompatibility compared to their bare counterparts, as demonstrated by the lifespan and locomotion assays.

Regarding the ultrasound treatment, the viability assay showed good biocompatibility until intensities higher than 2 W/cm^2^ for the treatment in water. On the other hand, in agar plates, body-bend assays performed after US stimulation showed an increase in the number of movements at 1 W/cm^2^ for 3 min and at 2 W/cm^2^ already for 1 min, demonstrating that the medium through which the mechanical wave propagates can influence the overall effect of the treatment.

The combined treatment indicated that mortality on *C. elegans* is primarily caused by ultrasound stimulation and its associated mechanical action. Regarding sublethal effects, the main impact was observed for the nanoparticles themselves, causing an increase in radical species and a decrease in the number of body bends. Overall, these results therefore show that there is no marked additive effect between ultrasound and nanoparticles on healthy animal models such as *C. elegans*. This consideration paves the way for a responsible and safe use of such stimuli-responsive nanosystems in contact with healthy tissues and organs and limits the possible therapeutic effects possibly to diseased tissues.

## Supplementary Material



## Data Availability

All data supporting the findings of this study are available within the paper and its Supporting Information. All raw data and uncropped blots are available upon request. Further information and requests for resources and reagents should be directed to and will be fulfilled by the lead contacts, Elia Di Schiavi (elia.dischiavi@cnr.it) and Valentina Cauda (valentina.cauda@polito.it).

## Notes

Additional schemes of experimental setup, viability data, and biodistribution images (PDF)
