# Supplementary material for: Toxicological and Physiological Responses to Combined Ultrasound and Lipid-Coated ZnO Nanoparticle Exposure in Caenorhabditis elegans
Source: ACS Appl Mater Interfaces. 2026 Jun 16;18(25):35030–41. doi: 10.1021/acsami.6c07340 (PMC13339014; doi:10.1021/acsami.6c07340)
Supplement: Supplementary file 1 [file am6c07340_si_001.pdf]

SUPPORTING INFORMATION

Toxicological And Physiological Responses to Combined Ultrasound and Lipid-coated ZnO Nanoparticle Exposure in *Caenorhabditis Elegans*

Elia Pascucci[1,§], Giorgia Savino[1,§], Pamela Santonicola[2], Giuseppina Zampi[2], Elia Di Schiavi[2,], Valentina Cauda[1,*]*

[1] Department of Applied Science and Technology, Politecnico di Torino, Corso Duca degli Abruzzi 24, 10129 Turin, Italy

[2] Institute of Biosciences and BioResources (IBBR), National Research Council of Italy (CNR), Via P. Castellino 111, 80131 Napoli, Italy

§E.P. and G.S. contributed equally to this work

*Corresponding authors: elia.dischiavi@cnr.it and valentina.cauda@polito.it

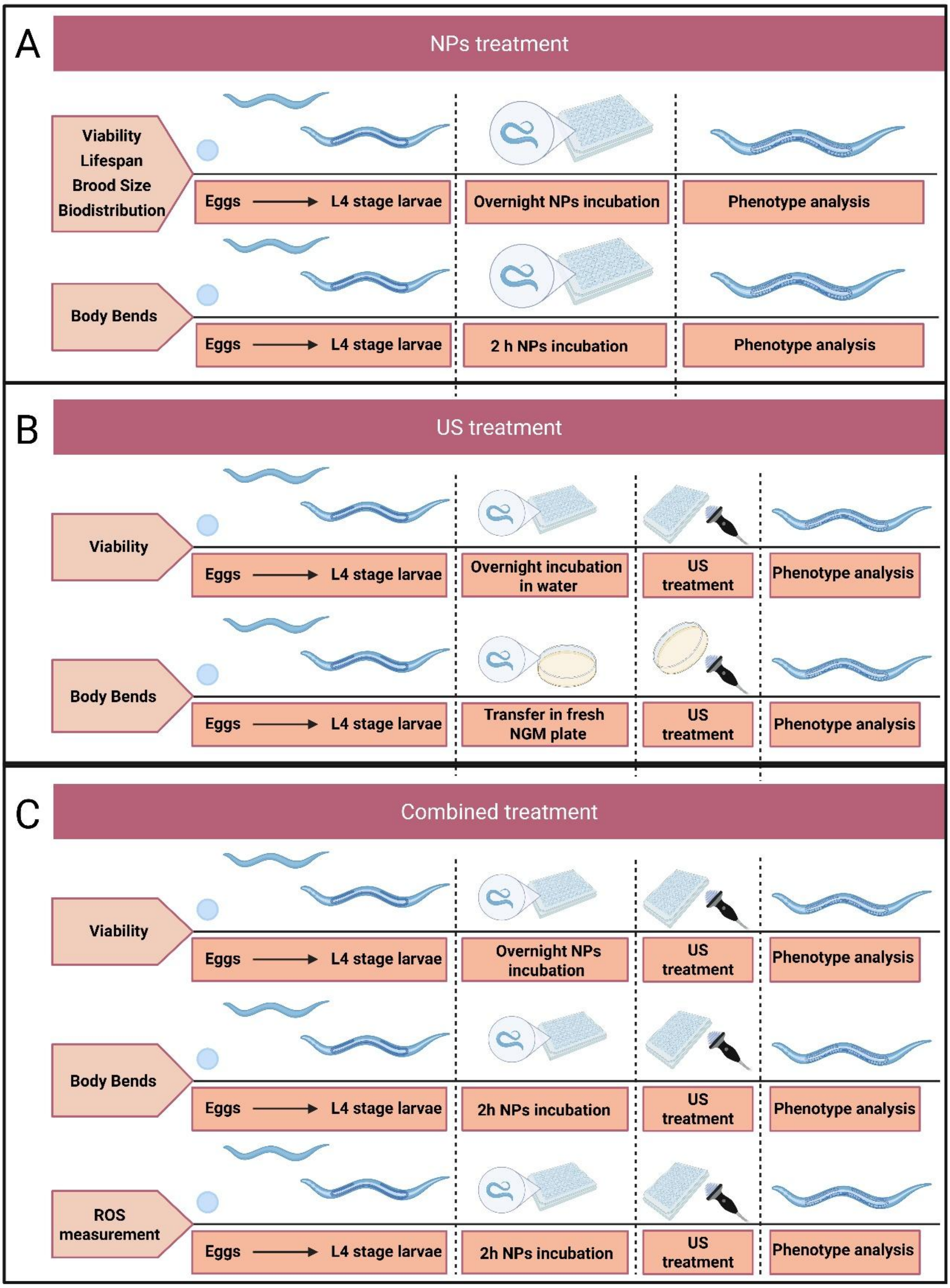

Figure S1. Scheme of the different treatments.

Timeline of treatments performed with (A) nanoparticles, (B) with ultrasound stimulation, and (C) combined treatment.

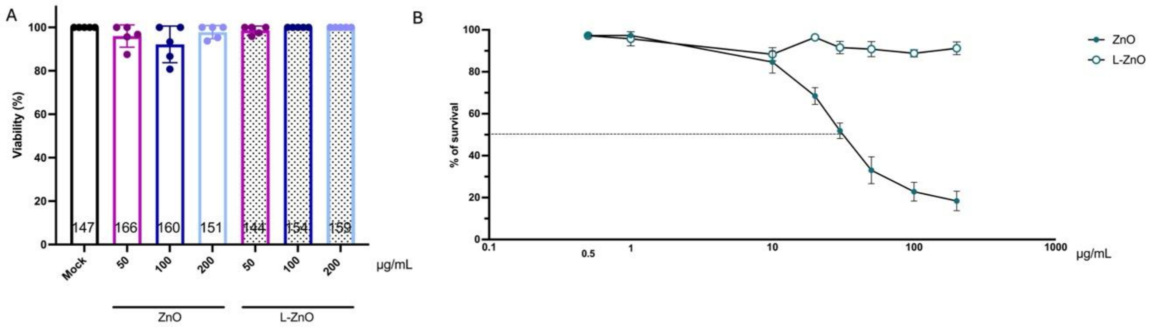

Figure S2. Viability after treatment with ZnO and L-ZnO NPs

(A) Quantification of the percentage of living animals after overnight exposure to ZnO and L-ZnO NPs (50, 100, 200 µg/mL), incubated with bacteria as food source. Exposure to NPs was performed from L4 larval stage animals. Each dot is the percentage of living animals for one well. Bar is the mean value. Error bars indicate SEM. (B) Quantification of the percentage of living animals after overnight exposure to ZnO and L-ZnO NPs (0.5, 1, 10, 20, 30, 50, 100, 200 µg/mL) in absence of bacteria as food source. Exposure to NPs was performed from L4 larval stage animals. Each dot is the mean percentage of live animals. Error bars indicate SEM.

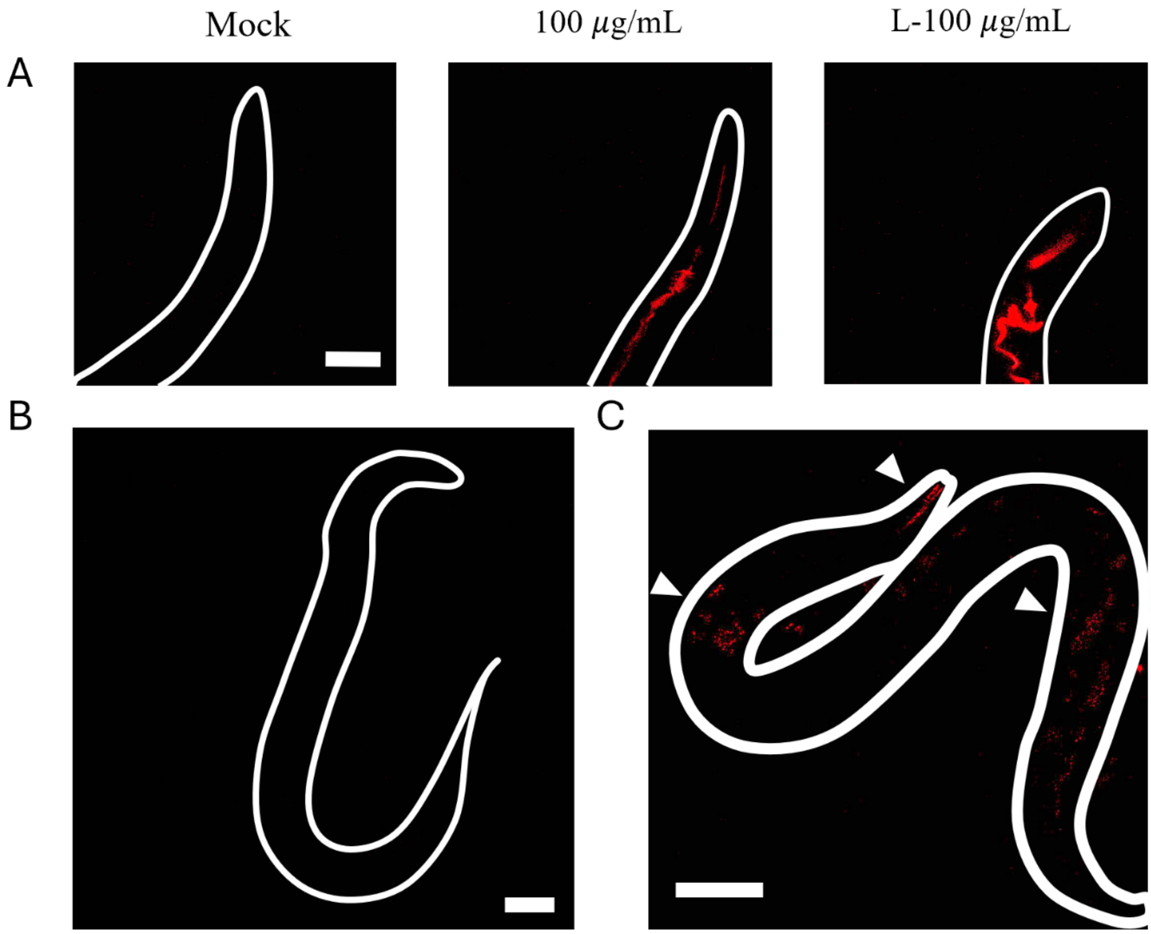

Figure S3. Biodistribution analysis after treatment with fluorescent ZnO and L-ZnO NPs.

(A) Images of the head of animals treated overnight with mock (left), labelled-ZnO (middle) and labelled-L-ZnO (right). In red ATTO-647 labelled-NPs. Scale bar is 100 µm. (B) Image of a whole animal treated overnight with mock. Scale bar is 50 µm. (C) Image of a whole animal treated overnight with labelled-L-ZnO. Arrowheads highlight the red signal of ATTO 647 labelled NPs. Head is up in all images. White lines correspond to the animal body. Scale bar is 50 µm.
